# Survey on Findings and Utilization of Preoperative Chest Radiography in Ophthalmic Surgery

**DOI:** 10.3390/jcm13133909

**Published:** 2024-07-03

**Authors:** Yohei Kuroki, Ayako Takamori, Koichiro Takahashi, Soichiro Yamamoto, Noriko Yoshida, Hiroshi Enaida

**Affiliations:** 1 Department of Ophthalmology, Faculty of Medicine, Saga University, 5-1-1 Nabeshima, Saga 849-8501, Japan; sx2780@cc.saga-u.ac.jp (Y.K.); stentorviolin@gmail.com (S.Y.); 2 Clinical Research Center, Saga University Hospital, Saga 849-8501, Japan; ayakotak@cc.saga-u.ac.jp (A.T.); nyoshida@cc.saga-u.ac.jp (N.Y.); 3 Division of Hematology, Respiratory Medicine and Oncology, Department of Internal Medicine, Faculty of Medicine, Saga University, Saga 849-8501, Japan; takahak@cc.saga-u.ac.jp

**Keywords:** chest radiography, preoperative examination, abnormal findings, interstitial shadow, ophthalmic surgery, perioperative complications, choosing wisely campaign

## Abstract

**Objective**: The objective of this paper is to reconsider the significance of preoperative chest radiography (CXR) before ophthalmic surgery through investigation of imaging findings and usage status. **Methods**: This retrospective observational clinical study involved 1616 patients who underwent ophthalmic surgery at Saga University Hospital from 1 January 2019 to 31 December 2020. The patients’ radiology reports were obtained from the electronic medical records, and their CXR findings, therapeutic interventions, and progress were investigated. **Results**: Among all patients, 539 (33.4%) had abnormal preoperative CXR findings. Of these patients, 74 (4.6%) had newly identified abnormal findings. In both patient groups, approximately 70% of patients with abnormal findings were aged ≥70 years, and interstitial shadows were the most common finding. Among all patients with abnormal findings, three (0.19%) received preoperative therapeutic interventions, and all surgeries were performed safely. Forty-three patients with abnormal findings were referred to our hospital or other hospitals for further investigation and treatment postoperatively. Among those patients, eight (0.5%) had primary lung cancer, seven underwent surgery, and one received chemoradiation. The other patients were also followed up and received appropriate therapeutic interventions. **Conclusions**: Before ophthalmic surgery, few patients required actual therapeutic interventions based on their CXR results. However, many abnormal findings were revealed in elderly patients, including some serious diseases. Furthermore, research has suggested that appropriate therapeutic intervention after ophthalmologic surgery may reduce the risk of a poor life prognosis. This study clearly shows that preoperative CXR is not only useful for perioperative systemic management but also ultimately benefits patients. It is also considered particularly meaningful for patients aged ≥70 years.

## 1. Introduction

Preoperative examinations are important to screen for preexisting conditions and unidentified diseases in patients undergoing surgery. Preoperative chest radiography (CXR) is used to evaluate the risk of respiratory or circulatory disorders and the presence or absence of respiratory infections such as tuberculosis. Preoperative CXR is essential if intraoperative general management with intubation by an anesthesiologist is required.

According to previous reports, preoperative CXR is recommended for patients with preoperative systemic complications such as heart disease, respiratory disease, a history of upper respiratory infection, and smoking [1,2]. However, one report indicated that preoperative examinations, including CXR, are unnecessary for patients undergoing cataract surgery because the surgery is minimally invasive and the examination does not lead to a reduction in perioperative surgical complications [3]. That report was reinforced by a subsequent review from a cost-effectiveness perspective [4]. By contrast, other reports have shown that approximately half (49–53%) of patients undergoing cataract surgery in the United States undergo preoperative examinations, including CXR, for various medical reasons [5,6].

At many facilities in Japan, including private clinics, cataract surgery is performed under local anesthesia on an outpatient basis. However, patients with severe eye diseases and high-risk patients with various underlying diseases and serious systemic complications are referred to large general hospitals, such as university hospitals and core hospitals. Many of these hospitals conduct preoperative screening tests such as blood tests, electrocardiography, and CXR before ophthalmic surgery, regardless of the target disease or type of anesthesia. Such screening tests are performed because many ophthalmic surgeries other than cataract surgeries such as vitrectomy are performed at these hospitals, and for particularly high-risk patients, preoperative examinations are performed to understand the patient’s general condition and prepare for sudden changes in the perioperative period. This allows the surgery to be more safely performed [7].

Therefore, what is the current state of obtaining detailed preoperative CXR findings for patients undergoing ophthalmic surgery at university hospitals? Furthermore, to what extent is perioperative intervention performed for patients with abnormal CXR findings? Even in patients who are considered healthy, unexpected abnormal findings may be discovered during preoperative examinations. In such cases, how are preoperative CXR results used beyond perioperative management? Are the results of preoperative CXR ultimately useful for patients? To answer these questions regarding preoperative CXR before ophthalmic surgery, we conducted a detailed investigation of the findings of preoperative CXR performed in our hospital. For patients with abnormal findings, we conducted a follow-up survey regarding subsequent treatment, including treatment in the perioperative period. Through these investigations, we clarified the actual state of preoperative CXR and reconsidered the significance of preoperative CXR from several perspectives.

## 2. Patients and Methods

This study involved 1616 consecutive patients who were admitted to Saga University Hospital from 1 January 2019 to 31 December 2020 and underwent ophthalmic surgery under local or general anesthesia after preoperative CXR. If the same patient was admitted multiple times, the patient was counted as one case.

For all patients, preoperative CXR was performed within 1 month before surgery. The CXR findings were obtained from the radiology report in the electronic medical record.

All findings were reported to the patients or their families before surgery. Furthermore, with the patient’s consent, we performed additional preoperative and postoperative examinations as necessary, and management of these findings was discussed with the attending physician.

When the surgery was considered affected by the preoperative CXR findings, appropriate measures were taken before or during surgery, and the scheduled surgery was performed. Patients with abnormal findings requiring postoperative therapeutic interventions were referred to the hospital of their choice, and their progress was confirmed. When the patient’s progress was unclear from materials such as medical charts and letters alone, the patient was directly examined to confirm their progress.

Previously identified abnormal CXR findings were defined as those that were identical to the current preoperative CXR findings based on CXR performed in the past at our hospital as well as information gathered from medical records and materials from other hospitals. Newly identified abnormal CXR findings were defined as those that had not been previously identified and were first confirmed on the current preoperative CXR. All abnormal CXR findings were defined as the sum of previously identified and newly identified abnormal CXR findings. All abnormal findings were counted, including cases where one patient had multiple abnormal findings.

In the statistical analysis, we presented patients characteristics and the distribution of all/newly identified abnormal findings as *n* (%) or mean ± standard deviation (SD), categorized by the target disease of ophthalmic surgery. Logistic regression analyses were performed to clarify the relationship of the presence or absence of abnormal findings with increasing age; the logistic regression analyses were performed, and odds ratios (ORs) and 95% confidence intervals (CIs) were calculated for each targeted disease. In addition, the chi-square tests were applied to investigate sex differences in abnormal CXR findings by age. JMP Pro 17.0.0 (SAS Institute Inc., Cary, NC, USA) was used for all analyses, and the statistical significance level was defined as *p* < 0.05.

## 3. Results

### 3.1. Patients’ Backgrounds and Surgically Treated Ophthalmic Diseases

The study population included 840 (52.0%) men and 776 (48.0%) women, totaling 1616 patients with a mean age of 66.6 ± 18.2 years (range, 2–97 years). The target diseases for ophthalmic surgery were cataracts (n = 787, 48.7%), retinal detachment (n = 160, 9.9%), epiretinal membrane (n = 100, 6.2%), glaucoma (n = 93, 5.8%), strabismus (n = 87, 5.4%), diabetic retinopathy (n = 65, 4.0%), macular hole (n = 64, 4.0%), intraocular lens dislocation (n = 35, 2.2%), ptosis (n = 33, 2.0%), pterygium (n = 20, 1.2%), and others (n = 172, 10.6%) (Table 1).

The mean age of patients with strabismus was 26.1 years, and the mean age of patients in the other disease groups ranged from 54.7 to 73.5 years (Table 1). Among patients who underwent surgery, 1469 (90.9%) received local anesthesia and 147 (9.1%) received general anesthesia. For those who underwent multiple surgical procedures simultaneously, only the main one disease was counted here.

### 3.2. Incidence of Abnormal CXR Findings before Ophthalmic Surgery

Preoperative abnormal CXR findings were observed in 539 (33.4%) of all 1616 patients. Among these 539 patients, 74 (4.6%) had newly identified abnormal findings and 465 (28.8%) had previously identified abnormal findings (Table 2). The investigation of abnormal findings and age showed that the presence of abnormal findings was significantly associated with older age (all diseases: OR, 1.06; *p* < 0.01) (Table 3).

Of the 539 patients with abnormal findings, 400 (74.2%) were aged ≥70 years and 139 (25.8%) were aged <70 years (Table 4). By disease, we found relationships between detection of abnormal findings and age for cataracts (OR, 1.07; *p* < 0.01), retinal detachment (OR, 1.06; *p* < 0.01), epiretinal membrane (OR, 1.08; *p* < 0.01), strabismus (OR, 1.08; *p* < 0.01), diabetic retinopathy (OR, 1.05; *p* = 0.049), macular hole (OR, 1.08; *p* = 0.03), ptosis (OR, 1.20; *p* = 0.03), and other diseases (OR, 1.05; *p* < 0.01) (Table 3). There were no sex-related differences in abnormal findings overall or by age group (Table 4). Five patients with newly identified abnormal CXR findings but no abnormalities on chest computed tomography (CT) were defined as having false-positive results; therefore, the sensitivity and specificity of preoperative CXR in ophthalmic surgery were 92.8% and 100%, respectively.

### 3.3. Abnormal Findings and Disease

Abnormal CXR findings in the total patient population were as follows: interstitial shadow (n = 229, 42.5%), cardiac enlargement (n = 207, 38.4%), nodular shadow (n = 57, 10.6%), pleural changes (n = 50, 9.3%), and others (n = 140, 26.0%). Regarding the relationship between all abnormal CXR findings and diseases, the top four diseases accounted for 77% of cases (Table 5). The newly identified abnormal CXR findings were interstitial shadows (n = 40, 54.1%), nodular shadows (n = 15, 20.3%), cardiac enlargement (n = 14, 18.9%), a mass (n = 9, 12.2%), and others (n = 15, 20.3%). Among these results, the difference from the overall newly identified abnormal findings is that the proportion of patients with a mass was high. No specific abnormal findings were detected by disease (Table 5).

### 3.4. Therapeutic Interventions in Patients with Abnormal CXR Findings and Perioperative Management of Ophthalmic Surgery

**Figure 1:**
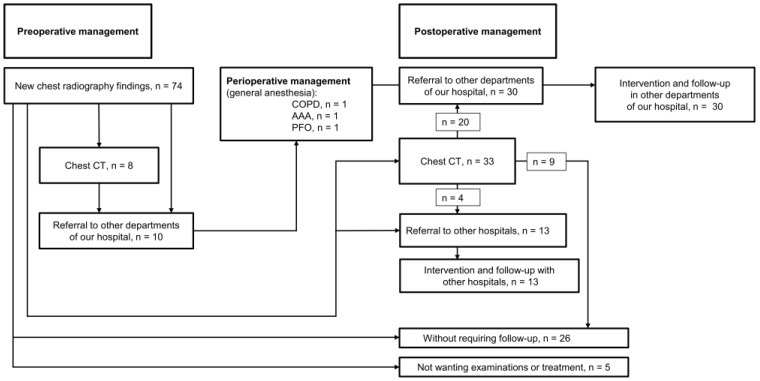
Management of 74 patients with new abnormal CXR findings.

**Figure 2:**
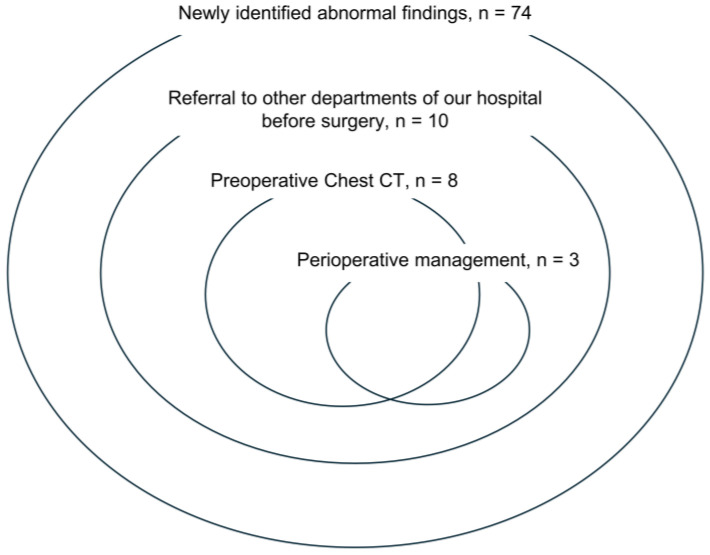
Venn diagram of preoperative intervention for 74 patients with newly identified abnormal findings.

Of the 465 patients with previously identified abnormal findings, 322 did not require follow-up as judged by the radiologists in our hospital. The remaining 143 patients underwent follow-up at other departments of our hospital or other hospitals. After checking with their attending physicians, we determined that there would be no problem with performing ophthalmic surgery. Of the 74 patients with newly identified abnormal findings, 10 patients were judged to have findings that would affect the ophthalmic surgery. Eight of the ten patients underwent CT and were referred to other departments of our hospital before surgery. Of these patients, perioperative therapeutic interventions were required in three, all of whom subsequently underwent surgery with general anesthesia (Figure 1 and Figure 2). Among these three patients, one patient with chronic obstructive pulmonary disease was scheduled to undergo cataract surgery under general anesthesia because of claustrophobia, and drug therapy was started preoperatively. To prevent perioperative deep vein thrombosis and thrombotic stroke during strabismus surgery under general anesthesia, pediatric patients with patent foramen ovale wore compression stockings during surgery. The patient who required vitrectomy and encircling because of retinal detachment (proliferative vitreoretinopathy) had an abdominal aortic aneurysm and required strict blood pressure control from preoperatively to the perioperative period (Table 6).

Of the 74 patients with newly identified abnormal CXR findings, 10 were referred to other departments of our hospital preoperatively. Of these patients, three required perioperative treatment. In addition to the preoperative referrals, 30 patients were referred to other departments of our hospital postoperatively. Thirteen patients were referred to other hospitals postoperatively. Appropriate intervention was made in all cases after referral, and follow-up was continued thereafter. Twenty-six patients were judged not to require follow-up, and five were recommended for further examination and follow-up but declined.

Of the 74 patients with newly identified abnormal findings, 8 underwent preoperative chest computed tomography (CT). Including these 8 patients, 10 patients were referred to other departments before surgery. Three of the patients referred to other departments required perioperative management (one of the three patients underwent preoperative chest CT).

In all cases, detailed examinations and therapeutic interventions to address the abnormal findings were performed after ophthalmic surgery. In all cases, including the three patients who received perioperative therapeutic interventions, surgery was performed as planned without the development of serious systemic complications during the perioperative period. Forty-three patients, including preoperative referrals, were referred to our hospital or other hospitals after ophthalmologic surgery for reexamination of abnormal findings and treatment. CT examinations were performed as necessary, and 30 patients (including 10 patients referred preoperatively) were referred to other departments of our hospital postoperatively (Figure 1). With the exception of 1 pediatric patient, 29 patients were aged >50 years, and 66.7% of these were aged >70 years. Among these 30 patients, 8 had primary lung cancer, 5 had non-tuberculous mycobacterial pulmonary disease, 3 had pleural plaques, 2 had thyroid tumors, 2 had abdominal aortic aneurysms, and 2 had heart failure. One patient each had chronic obstructive pulmonary disease, patent foramen ovale, old pulmonary tuberculosis, sarcoidosis, pulmonary aspergillosis, pulmonary cryptococcosis, obliterative hypertrophic cardiomyopathy, and a pancreatic cyst. Although the pancreatic cyst was unrelated to the CXR findings, the diagnosis was made by detailed CT. The most common cause of interstitial shadows that required detailed investigation was non-tuberculous mycobacterial pulmonary disease.

Seven of the eight patients with primary lung cancer underwent ophthalmic surgery followed by surgery, and one received chemoradiotherapy. One of the patients with a thyroid tumor underwent surgery after ophthalmologic surgery. Another patient had a small thyroid tumor, but because it was not a functional tumor, it was not indicated for treatment and was instead followed up. Patients with abdominal aortic aneurysm, sarcoidosis, and hypertrophic cardiomyopathy obliterans received oral medications after ophthalmic surgery. Other diseases were followed up at the referral department (Table 6).

Thirteen patients were referred to other hospitals postoperatively. Except for one patient who was 67 years old, the remaining 12 patients were all >70 years old. The 13 patients comprised 6 patients with interstitial pneumonia, 4 patients with partial atelectasis, and 1 patient each with hypersensitivity pneumonitis, allergic bronchopulmonary aspergillosis, and pleural plaque. Among these, drug treatment was started for the patients with interstitial pneumonia, and follow-up was conducted for the other patients (Table 7).

Thirty-one patients were not referred. Of these patients, 26 did not require follow-up as judged by the radiologists, who based their decision on the CXR or CT results (17 patients with old inflammatory changes, 5 without abnormal findings on CT, 2 with hiatal hernias, and 1 each with chronic airway inflammation and subcutaneous fatty tissue). Detailed examination and follow-up were recommended for the remaining five patients; however, they declined this follow-up (Figure 1). Therefore, among all patients, perioperative intervention was considered in 10 (0.62%), and 3 (0.19%) actually required intervention. In addition, 43 (2.66%) patients required therapeutic intervention (including preoperative intervention) or follow-up observation after ophthalmic surgery as determined by the attending physician. All of these patients were those with newly identified abnormal findings.

## 4. Discussion

In this study, we conducted a detailed investigation of the content of preoperative CXR for ophthalmic surgery. Few detailed reports to date have focused on preoperative CXR findings. However, these few reports contain some interesting findings. Thirty years ago, a preoperative CXR survey before ophthalmic surgery conducted at a local university in Japan revealed abnormal findings in 35.8% of patients, mainly elderly patients, and 4.1% of these were newly identified abnormal findings [8]. This rate is identical to that in our study. The most common among the abnormal findings was “pulmonary fibrosis and calcification,” which accounted for 57.9% of the abnormal findings. The cause was considered to be the influence of pulmonary tuberculosis, which had been prevalent since before World War II [8]. The differences in findings and causative diseases between that study and ours are thought to be due to differences in the historical backgrounds.

A recent study also examined CXR findings before cardiac surgery. Abnormal findings were observed in half of the patients preoperatively, with cardiomegaly and aortic elongation being the main abnormal findings [9]. This suggests that differences in preoperative CXR findings are associated with differences in the surgical target.

CXR is performed as part of mandatory workplace annual health examinations in Japan for workers aged 20 to 64 years. In a survey of 8,594,676 people, the percentage of abnormal findings was 9.1% for men and 6.7% for women, and the percentage of abnormal findings was found to increase with age [10]. The significantly higher rate of abnormal findings in the preoperative examination mentioned above compared with the rate in this annual health examination may be explained by the fact that the target age group was older in the preoperative examination and that many patients had systemic complications.

As the target age, period, and disease targeted for surgery change, the rate of abnormal CXR findings and the content of these findings will also change. Investigations based on the disease targeted for surgery are important because the measures required in the perioperative period are likely to change accordingly.

With respect to perioperative therapeutic interventions based on findings related to the significance of preoperative CXR, ten patients with newly identified findings consulted with other departments before surgery, and three of them were treated under appropriate perioperative management. The intervention rate was 0.19% in this study. Furthermore, in the previous reports mentioned above, the intervention rates were 0.2% [8] and 0.8% [9].

Overall, previous reports (including our report) have shown that the number of perioperative therapeutic interventions is lower than the number of preoperative CXR examinations performed. Thus, the need for preoperative examinations generally ends once the intended surgery has been successfully completed. As previously reported, it is generally considered that preoperative CXR is not necessary, especially when there are few opportunities for perioperative intervention (such as in minimally invasive cataract surgery) [3,4].

From a non-medical perspective, preoperative CXR examinations for cataract surgery are undesirable because of the increased medical cost. In the United States, more than 4.4 million cataract surgeries are expected to be performed by 2030, and excessive preoperative examinations are seen as a major problem because of concerns about the impact they may have on the insurance system [5]. More than $30 billion is spent annually in the United States on preoperative examinations for all surgeries [11]. Therefore, health authorities in the United States have advocated minimizing preoperative examinations for cataract surgery from a cost-effectiveness perspective [3,4]. The Choosing Wisely Campaign, which was launched in the United States in 2012 to avoid unnecessary medical examinations and treatments and effectively utilize medical resources, is spreading worldwide, including within the field of ophthalmology [6,12].

Despite these proposals and campaigns, preoperative testing, including CXR, is still performed on many patients in the United States even during cataract surgery [5,6]. This is occurring because patients undergoing ophthalmic surgery are not only low-risk patients; this population also includes high-risk patients because of differences in medical systems and facilities. Additionally, general hospitals tend to conduct more tests than clinics, and differences in individual physicians’ practice patterns are reportedly one of the reasons for this [5,6]. In ophthalmic surgery in Japan, high-risk patients who are judged unsuitable for surgery at a clinic, regardless of their age, are usually referred to large general hospitals (such as university hospitals or core hospitals) for treatment. Physicians at these facilities, who often handle difficult cases, generally believe that preoperative examinations, including CXR, are an important part of the perioperative evaluation to ensure safe surgery.

Many surgeries other than cataract surgery are performed at university hospitals (Table 1), and many of these patients are in poor general condition. Among the various systemic complications encountered in the perioperative period of vitrectomy, reports indicate occurrences such as postoperative acute pulmonary edema [13], which is likely to be caused by ischemic heart disease, and mild ketoacidosis in 20% of patients following surgery for diabetic retinopathy. There are also reports of myocardial infarction and difficulty urinating [14].

In the present study, abnormal findings related to the respiratory and cardiovascular systems were often observed during ophthalmic surgery. Even for patients not undergoing general anesthesia, preoperative examinations, including CXR, may be required for perioperative management depending on the nature of the surgery or systemic disease. Many preoperative tests are actually performed reflexively in anticipation of complications. The costs associated with preoperative CXR are low and easily compensated if the information provided by preoperative CXR prevents unnecessary surgery and prolonged hospital stays. Large-scale studies are needed to evaluate the significance of preoperative examinations for various surgeries, taking into consideration the increasing medical costs. The suitability of preoperative examinations may need to be reconsidered in many cases.

After perioperative management, how should the preoperative CXR results be handled? In previous reports [3,4,5,6,8,9], follow-up studies after the perioperative period were not performed. The present study also included a follow-up survey after perioperative risk management based on the preoperative CXR findings.

Preoperative CXR is a medical procedure performed with the patient’s consent. In Japan, it is considered important that even after the perioperative period, the results of preoperative CXR are ultimately beneficial to the patient. If the results of examinations are overlooked and the patient is seriously disadvantaged, even if the results have no association with the intended surgery, the doctor in charge will be held accountable and the medical facility will be exposed to social criticism [15]. Therefore, at hospitals in Japan, preoperative CXR findings are properly evaluated by multiple doctors, including the attending physician, and any necessary actions are implemented through appropriate medical coordination. Hospitals undoubtedly require a system to prevent examination results from being overlooked.

In this study, 43 (2.7%) patients with abnormal CXR findings underwent detailed examinations before and after ophthalmic surgery. Of the 43 patients, 42 were aged >50 years. Of these 42 patients, 30 underwent detailed examination, treatment, and follow-up observation before and after ophthalmic surgery at our hospital. These included eight patients with primary lung cancer, two with abdominal aortic aneurysms, two with thyroid tumors, and one with hypertrophic obstructive cardiomyopathy, which would be fatal if left untreated.

Further examination of the frequently observed interstitial shadows in this study revealed that non-tuberculous mycobacterial pulmonary disease was the most common cause. All 30 of these patients were asymptomatic during preoperative examination, and physical examination was not predictive of their illnesses. In addition, appropriate examinations, treatment, and follow-up observations were performed at the referral destination for 13 patients who were referred to a hospital other than ours. As a result, patients who continued treatment had extremely high levels of satisfaction with their treatment, including the initial ophthalmic surgery.

Notably, the detection rate for primary lung cancer in this survey was 0.5%. The detection rate in the mandatory workplace annual health examinations in Japan is 0.0091% [10], and that in the lung cancer screening in Japan for people aged >40 years is 0.098% for men and 0.047% for women [16]. Therefore, the detection rate of primary lung cancer using CXR before ophthalmology surgery is extremely high compared with the rate in official screenings conducted in Japan. This result was an unexpected finding of our study.

In Japan, CXR is mandatory for workers aged ≥40 years as a part of workplace annual health examinations. However, CXR is not performed on retired workers aged ≥70 years [10]. Therefore, the positive rate of lung cancer in workplace annual health examinations is considered low. The lung cancer screening program in Japan involved 1,030,119 people in 2016, among whom 42.1% were aged ≥70 years. The lung cancer detection rate increases with age; it reached 0.130% (0.21% for men and 0.057% for women) in the 2016 cohort of people aged ≥80 years [16], which is lower than the positive rate in the present study. In this study, we found that the proportion of patients aged ≥70 years among all patients was 53.0%, approximately 10% higher than in lung cancer screening. However, beyond the age of the patients, other contributing factors to the difference in the lung cancer detection rate remain unknown.

Unfortunately, we were unable to investigate the patient background factors that may lead to lung cancer. However, possible reasons for the difference in the positive rate of lung cancer may include slight differences in the age of the patients as well as the presence or absence of underlying diseases between these studies. Although further studies are needed, preoperative CXR before ophthalmic surgery may complement the lung cancer screening findings in patients aged ≥70 years.

This study of preoperative CXR revealed abnormal findings in one-third of patients, mainly elderly patients. Although preoperative CXR is often considered a necessary examination for preoperative evaluation and perioperative management to ensure safe surgery, only a few patients required perioperative intervention as a result of the CXR findings. However, as shown in this study, preoperative CXR may reveal abnormal findings that the patient did not notice because they were asymptomatic. This is especially true in elderly patients, and it occurred at a higher rate than in workplace examinations and cancer screenings. For patients with findings that require further examinations, the lifetime prognosis can be improved by performing appropriate therapeutic interventions after ophthalmic surgery. Additionally, cost-effectiveness issues may be overcome if test results are properly communicated to patients and used effectively. Furthermore, use of such a comprehensive medical response system may be one of the reasons why the average life expectancy in Japan has been extended.

Given the expansion of the Choosing Wisely Campaign and the results of this study, young patients may not require CXR before ophthalmic surgery unless they plan to undergo general anesthesia. Through this study, it became clear that many of the patients who underwent preoperative CXR had previously identified abnormal findings, and most of these abnormal findings were already being properly monitored by the patients’ attending physicians. Therefore, we felt the need to make efforts to reduce unnecessary testing as much as possible for patients aged <70 years, such as by utilizing previous CXR data during regular monitoring by their attending physicians. At the same time, it became clear that the positive rate, including newly identified abnormal findings, was extremely high at approximately 70% in patients aged ≥70 years compared with other age groups. Therefore, CXR before ophthalmologic surgery is considered meaningful for older patients, especially those aged ≥70 years, in institutions such as university hospitals that manage severe cases because it offers potential future benefits for the patients and safer perioperative management.

## 5. Limitations

This study has two main limitations. First, both this study and a previous report [8] were conducted at a university hospital within a local city in Japan. Therefore, the results may differ from those in facilities in urban areas such as the metropolitan area. Second, there may be subjectivity in the interpretation of the CXR findings by different radiologists. In this study, the CXR findings were dependent on individual radiologist-reported results. Interpretation of radiographic images may vary depending on the radiologist’s experience, expertise, and personal judgment, which can lead to variability in the assessment and affect the accuracy and consistency of the data. With the introduction of artificial intelligence technology, it is likely that variations in interpretations will be reduced in the future. This is expected to lead to reduced variation in results and lower workloads.

## 6. Conclusions

Before ophthalmic surgery, few patients required actual therapeutic interventions based on their CXR results. However, many abnormal findings were revealed in elderly patients, including some serious diseases. Furthermore, research has suggested that appropriate therapeutic intervention after ophthalmologic surgery may reduce the risk of a poor life prognosis. This study clearly shows not only that preoperative CXR is useful for perioperative systemic management but also ultimately benefits patients. It is also considered particularly meaningful for patients aged ≥70 years.

## Figures and Tables

**Table 1:** Characteristics of patients by target disease for ophthalmic surgery.

Target Disease for Ophthalmic Surgery	n (%)		Age in Years, Mean ± SD (Range)		Sex, M/F
All diseases	1616	(100)	66.6 ± 18.2	(2–97)	840/776
Cataract	787	(48.7)	73.5 ± 11.6	(5–97)	415/372
RD	160	(9.9)	54.7 ± 16.0	(10–88)	56/104
ERM	100	(6.2)	68.9 ± 8.2	(46–86)	45/55
Glaucoma	93	(5.8)	68.1 ± 15.8	(16–96)	34/59
Strabismus	87	(5.4)	26.1 ± 23.1	(2–86)	44/43
DR	65	(4.0)	61.3 ± 12.1	(37–88)	22/43
MH	64	(4.0)	68.5 ± 9.8	(30–89)	39/25
IOL dislocation	35	(2.2)	67.2 ± 14.1	(37–89)	10/25
Ptosis	33	(2.0)	69.2 ± 13.3	(22–83)	20/13
Pterygium	20	(1.2)	60.7 ± 14.6	(31–78)	8/12
Others	172	(10.6)	65.6 ± 20.6	(6–95)	147/25

Abbreviations: SD, standard deviation; F, female; M, male; RD, retinal detachment; ERM, epiretinal membrane; MH, macular hole; DR, diabetic retinopathy; IOL, intraocular lens.

**Table 2:** Distribution of abnormal CXR findings by target disease for ophthalmic surgery.

Target Disease for Ophthalmic Surgery	Patients Examined, n	Abnormal CXR Findings			
		All Abnormal Findings, n (%) ^1^, (%) ^2^		Newly Identified Abnormal Findings, n (%) ^1^, (%) ^2^	
All diseases	1616	539	(33.4), (33.4)	74	(4.6), (4.6)
Cataract	787	317	(19.6), (40.1)	37	(2.3), (4.7)
Retinal detachment	160	33	(2.0), (20.6)	5	(0.3), (3.1)
Epiretinal membrane	100	32	(2.0), (32.0)	6	(0.4), (6.0)
Glaucoma	93	30	(1.9), (32.3)	5	(0.3), (5.4)
Strabismus	87	7	(0.4), (8.0)	2	(0.1), (2.3)
Diabetic retinopathy	65	21	(1.2), (32.3)	2	(0.1), (3.1)
Macular hole	64	19	(1.2), (29.7)	5	(0.3), (7.8)
IOL dislocation	35	11	(0.7), (31.4)	2	(0.1), (5.7)
Ptosis	33	10	(0.6), (30.3)	0	(0.0), (0.0)
Pterygium	20	4	(0.2), (20.0)	1	(0.1), (5.0)
Others	172	55	(3.4), (32.0)	9	(0.6), (5.2)

All abnormal findings: newly identified abnormal findings + previously identified abnormal findings. Newly identified abnormal findings: new abnormal findings noticed for the first time by the preoperative CXR before ophthalmologic surgery at our hospital. Previously identified abnormal findings: abnormal findings observed at other hospitals or our hospital (including other medical departments) in the past. Abbreviations: CXR, chest radiography; IOL, intraocular lens. ^1^ Percentage of patients with abnormal findings among all eligible patients. ^2^ Percentage of patients with abnormal findings among patients with each target disease.

**Table 3:** Relationship between abnormal findings and age by targeted disease for ophthalmic surgery.

Targeted Disease for Ophthalmic Surgery	OR	95% CI	*p* Value
All diseases	1.06	1.05–1.07	<0.01
Cataract	1.07	1.05–1.09	<0.01
RD	1.06	1.02–1.09	<0.01
ERM	1.08	1.02–1.14	<0.01
Glaucoma	1.03	1.00–1.06	0.09
Strabismus	1.06	1.02–1.10	<0.01
DR	1.05	1.00–1.10	0.049
MH	1.08	1.01–1.16	0.03
IOL dislocation	1.02	0.97–1.08	0.41
Ptosis	1.2	1.02–1.40	0.03
Pterygium	1.24	0.96–1.60	0.11
Others	1.05	1.02–1.07	<0.01

The *p* values were obtained by logistic regression analysis. Abbreviations: OR, odds ratio; CI, confidence interval; RD, retinal detachment; ERM, epiretinal membrane; MH, macular hole; DR, diabetic retinopathy; IOL, intraocular lens.

**Table 4:** Distribution of sex-related differences in abnormal CXR findings by age.

Age, Years	Sex	Patients Examined, n	Patients with Abnormal Findings, n (%)		Percentage of Patients with Abnormal Findings in Each Age Group by Sex, %	*p* Value
<50	M	123	10	(0.6)	8.1	0.69
	F	91	6	(0.4)	7.0	
50–59	M	105	17	(1.1)	16.2	0.51
	F	74	9	(0.6)	12.2	
60–69	M	210	58	(3.6)	27.6	0.67
	F	156	39	(2.4)	25.0	
70–79	M	238	101	(6.3)	42.4	0.97
	F	245	99	(6.1)	40.4	
≥80	M	164	88	(5.4)	53.7	0.81
	F	210	112	(6.9)	53.3	
Total	M	840	274	(17.0)	32.6	0.64
	F	776	265	(16.4)	34.1	
	M + F	1616	539	(33.4)	33.4	

Abbreviations: CXR, chest radiography; F, female; M, male. *p* values were obtained by the chi-square test.

**Table 5:** Abnormal CXR findings and incidence by target disease for ophthalmic surgery.

Abnormal CXR Findings All Abnormal Findings /Newly Identified Abnormal Findings	Target Diseases for Ophthalmic Surgery																							
	All Diseases		Cataract		RD		ERM		Glaucoma		Strabismus		DR		MH		IOL Dislocation		Ptosis		Pterygium		Others	
Interstitial shadow	229	/40	137	/24	14	/2	19	/4	14	/2	3	/1	7	/1	8	/2	9	/2	4	/0	0		14	/2
Cardiac enlargement	207	/14	120	/8	14	/1	17	/1	11	/0	5	/1	14	/0	4	/0	4	/0	5	/0	2	/0	11	/3
Nodular shadow	57	/15	33	/6	1	/0	2	/1	6	/3	1	/1	0		4	/1	0		1	/0	1	/1	8	/2
Pleural changes	50	/5	16	/3	5	/0	5	/0	3	/0	3	/0	5	/0	0		1	/0	0		0		12	/2
Pleural effusion	29	/4	17	/4	1	/0	1	/0	0		0		0		1	/0	1	/0	1	/0	0		7	/0
Calcification	27	/0	14	/0	3	/0	0		1	/0	0		3	/0	1	/0	0		1	/0	1	/0	3	/0
Emphysematous change	24	/0	12	/0	2	/0	0		2	/0	0		0		0		0		0		0		8	/0
Bone fracture	18	/0	10	/0	0		2	/0	1	/0	0		1	/0	0		0		1	/0	0		3	/0
Mass	12	/9	4	/3	2	/2	0		0		0		1	/1	3	/3	0		0		0		2	/0
Enhanced vascular shadows	10	/2	10	/2	0		0		0		0		0		0		0		0		0		0	
Hiatal hernia	7	/0	7	/0	0		0		0		0		0		0		0		0		0		0	
Bronchiectasis	5	/1	2	/1	0		0		0		0		0		0		0		0		0		3	/0
Bilateral hilar lymphadenopathy	3	/2	2	/1	0		0		0		0		0		0		0		0		0		1	/1
Atelectasis	2	/0	0		2	/0	0		0		0		0		0		0		0		0		0	
Aortic aneurysm	2	/1	1	/1	0		0		0		1	/0	0		0		0		0		0		0	
Situs inversus	1	/0	1	/0	0		0		0		0		0		0		0		0		0		0	

Some patients had multiple abnormal findings. Abbreviations: CXR, chest radiography; RD, retinal detachment; ERM, epiretinal membrane; MH, macular hole; DR, diabetic retinopathy; IOL, intraocular lens.

**Table 6:** Details of patients referred to other departments in our hospital.

Age, Years	Sex	Targeted Disease for Ophthalmic Surgery	Abnormal Findings of CXR	Final Diagnosis after Detailed Examination
6	M	Strabismus	Cardiac enlargement	PFO *
52	M	Glaucoma	Nodular shadow	Thyroid tumor
56	M	Glaucoma	Nodular shadow	LC
57	M	ERM	Interstitial shadow	Pleural plaque
61	F	Cataract	Enhanced vascular shadows	COPD *
61	M	RD	Cardiac enlargement	Heart failure
61	M	RD	Mass	Thyroid tumor
63	M	Cataract	Mass	LC
65	M	Others	Cardiac enlargement	HOCM
67	M	Others	Interstitial shadow	Pleural plaque
70	M	Cataract	Mass	LC
70	M	Cataract	Nodular shadow	Pancreatic cysts and old inflammatory changes
70	M	Cataract	Interstitial shadow	Pulmonary aspergillosis
71	F	Cataract	Interstitial shadow	NTM pulmonary disease
72	F	Cataract	Bilateral hilar lymphadenopathy	Sarcoidosis
73	F	Cataract	Interstitial shadow	NTM pulmonary disease
73	M	DR	Interstitial shadow	NTM pulmonary disease
76	F	Cataract	Interstitial shadow	Old pulmonary tuberculosis
77	F	Pterygium	Nodular shadow	LC
82	M	Cataract	Interstitial shadow	Pulmonary cryptococcosis
82	F	Cataract	Interstitial shadow	LC
83	M	Cataract	Interstitial shadow	NTM pulmonary disease
83	F	Cataract	Mass	LC
86	F	MH	Mass	LC
86	F	Glaucoma	Nodular shadow	LC
86	M	RD	Mass	AAA *
86	M	Others	Cardiac enlargement	AAA
86	F	Glaucoma	Interstitial shadow	NTM pulmonary disease
87	F	Cataract	Cardiac enlargement	Heart failure
93	M	Cataract	Pleural changes	Pleural plaque

* Patients requiring intervention before ophthalmic surgery. Abbreviations: CXR, chest radiography; M, male; F, female; RD, retinal detachment; ERM, epiretinal membrane; MH, macular hole; DR, diabetic retinopathy; PFO, patent foramen ovale; LC, lung cancer; COPD, chronic obstructive pulmonary disease; HOCM, hypertrophic obstructive cardiomyopathy; NTM, non-tuberculous mycobacterial; AAA, abdominal aortic aneurysm.

**Table 7:** Details of patients referred to other hospitals.

Age, Years	Sex	Targeted Disease for Ophthalmic Surgery	Abnormal Findings of CXR	Final Diagnosis after Detailed Examination
67	M	Cataract	Interstitial shadow	Hypersensitivity pneumonitis
70	M	Cataract	Interstitial shadow	Pulmonary aspergillosis
73	F	Cataract	Interstitial shadow	IP
73	M	DR	Mass	Partial atelectasis
74	F	Cataract	Interstitial shadow	IP
76	F	Cataract	Interstitial shadow	IP
81	F	Cataract	Interstitial shadow	IP
81	F	Cataract	Interstitial shadow	IP
81	M	ERM	Interstitial shadow	IP
83	M	MH	Mass	Partial atelectasis
83	M	MH	Mass	Partial atelectasis
87	F	Cataract	Bronchiectasis	Partial atelectasis
93	M	Cataract	Pleural changes	Pleural plaque

Abbreviations: CXR, chest radiography; M, male; F, female; ERM, epiretinal membrane; MH, macular hole; DR, diabetic retinopathy; IP, interstitial pneumonia.

## Data Availability

The data presented in this study are available on request from the corresponding author due to extracting data from patient records.
